# F158. FUNCTIONAL CONNECTIVITY DIVERSITY OF THE INSULA CORTEX IN SCHIZOPHRENIA: SUBREGIONS OR CONTINUA?

**DOI:** 10.1093/schbul/sby017.689

**Published:** 2018-04-01

**Authors:** Ye Tian, Chad Bousman, Chenxing Liu, Christos Pantelis, Andrew Zalesky

**Affiliations:** 1 The University of Melbourne; 2 University of Calgary; 3 The University of Melbourne, Melbourne Neuropsychiatry Centre

## Abstract

**Background:**

The function, cytoarchitecture and connectivity of the insula cortex are diverse. Cluster analyses have been applied to functional magnetic resonance imaging (MRI) connectivity data to parse this diversity and subdivide the insula into discrete subregions. However, the number of subregions comprising the insula remains vexed and whether these putative subregions are disturbed in neuropsychiatric illness are unknown. The present study aimed to (i) rigorously evaluate the number of subregions (if any) into which the insula can be subdivided based on topographic variation in whole-brain patterns of insula functional connectivity; and, (ii) establish whether the connectional topography of the insula is altered in schizophrenia.

**Methods:**

Two alternative models explaining the heterogeneity of insula connectivity were tested: (Model i) insula comprising discrete subregions, each associated with a distinct connectivity fingerprint; and, (Model ii) connectivity varying as a continuum across insula, without marked boundaries. Cluster analysis was used to delineate discrete subregions, and a novel gradient-based method was developed to evaluate whether connectivity varied continuously across the insula. These models were tested in a sample of individuals with schizophrenia (N=49), healthy comparison individuals (N=52) and an independent validation cohort from the Human Connectome Project (N=50).

**Results:**

Cluster analyses indicated that the insula comprised anterior and posterior subregions, with significantly less differentiation in connectivity patterns between these two clusters in the schizophrenia group (right: P=.0038; left: P=.002). The anterior insula was more strongly connected to the sensory-motor, occipital/parietal cortex and posterior lobe of cerebellum in the schizophrenia group, whereas the connectivity between the posterior insula and prefrontal cortex and thalamus was stronger in the patients (PFWE<.05). The dysconnectivity between anterior insula and anterior cingulate cortex was correlated with the severity of emotion withdrawal (Jonckeere-Terpstra test; JT=-3.74, P<.001). Most importantly however, for the majority of individuals in both datasets, the degree of cluster separation between insula subregions identified with cluster analyses was not significantly improved compared to clusters delineated in null data that was generated from white matter, where no clusters were expected. Modeling patterns of insula connectivity as continua of variation across a rostrocaudal axis was found to provide a more parsimonious model than using distinct subregions segregated by sharp boundaries. The variation in connectivity across this rostrocaudal axis was significantly reduced in schizophrenia patients (P=0.02).

**Discussion:**

This is the first study that comprehensively investigate the potential differences in connectional pathology of insula between its anterior and posterior aspects. We conclude that the connectional diversity of the insula inferred from resting-state functional connectivity should be conceptualized as continua of variation, rather than discrete subregions. We posit that the reduced differentiation between the anterior and posterior insula in schizophrenia may impact on the ability in discriminating self-generated from externally-generated sensory information, possibly contributing to hallucinations in the disorder.

